# Supplementary material for: ROS produced by Dual oxidase regulate cell proliferation and haemocyte migration during leg regeneration in the cricket
Source: Development. 2025 Nov 24;152(22):dev204763. doi: 10.1242/dev.204763 (PMC12687331; doi:10.1242/dev.204763)
Supplement: Supplementary information [file develop-152-204763-s1.pdf]

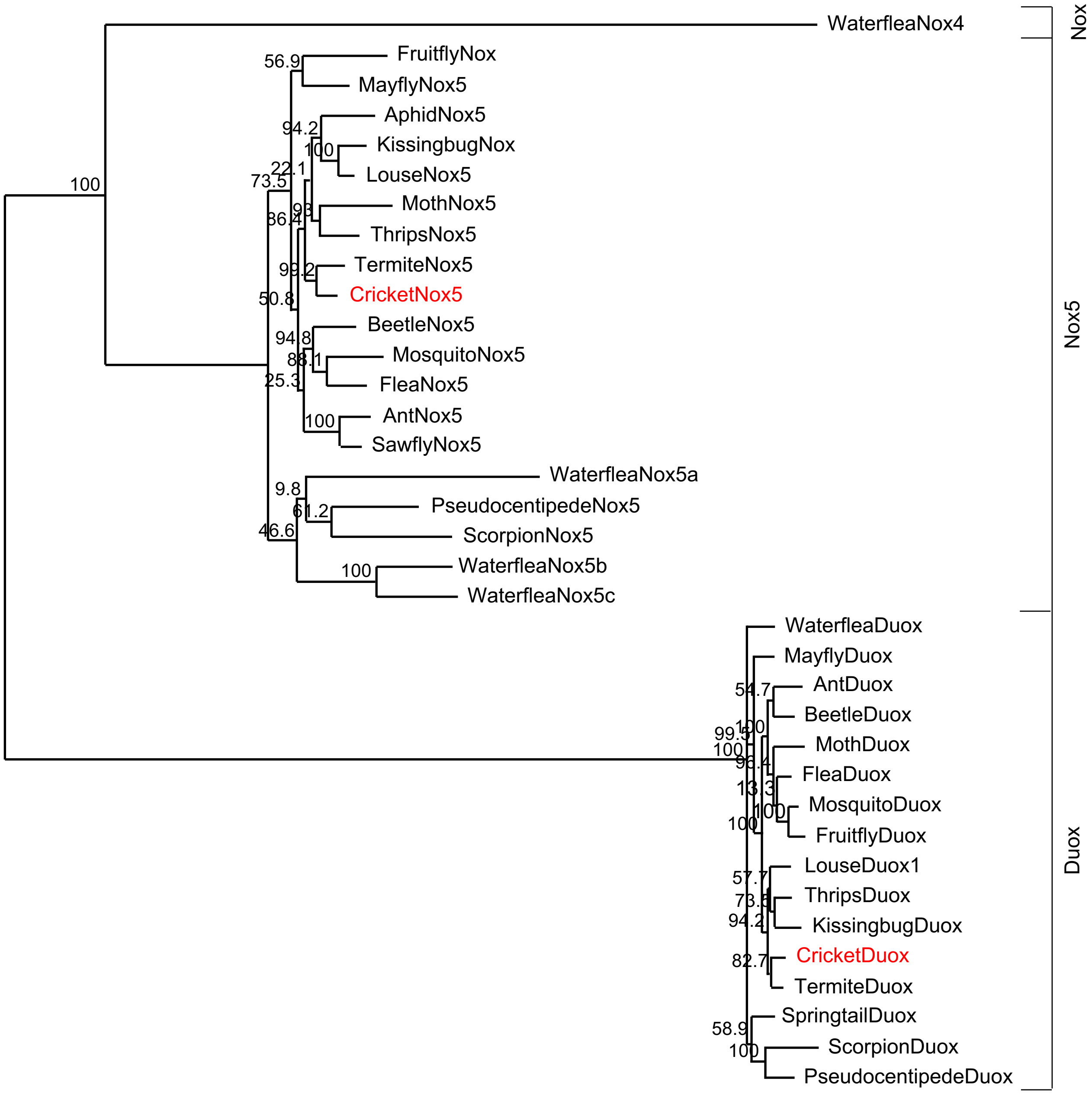

Fig. S1. Phylogenetic tree of NAPDH oxidases.

Phylogenetic tree of Duox, Nox5, and Nox of *Gryllus bimaculatus* and other arthropods based on amino acid sequence alignment using CLUSTALW.

Development • Supplementary information

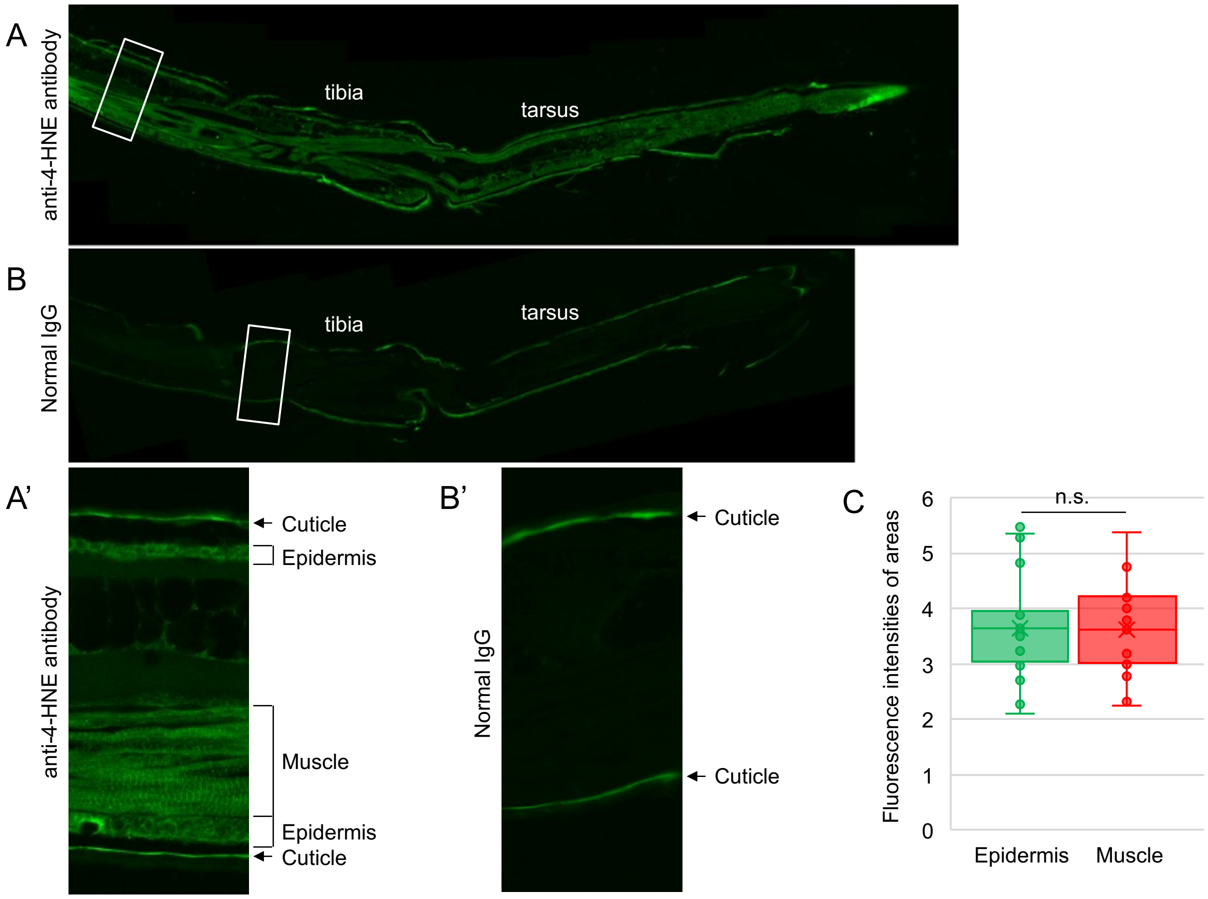

Fig. S2. Reactive oxygen species (ROS) detection in unamputated leg.
(A, A') ROS detection using an anti-4-HNE antibody in unamputated leg. (B, B) Negative control experiments using normal IgG. (A, B) Low-magnification images of the distal half of the tibia and tarsus. (A', B') High magnification image of square region of (A) and (B). (C) Fluorescence intensities in the epidermis and muscle of the unamputated leg were measured using the ImageJ software and are shown in the box plot. n.s., not significant.

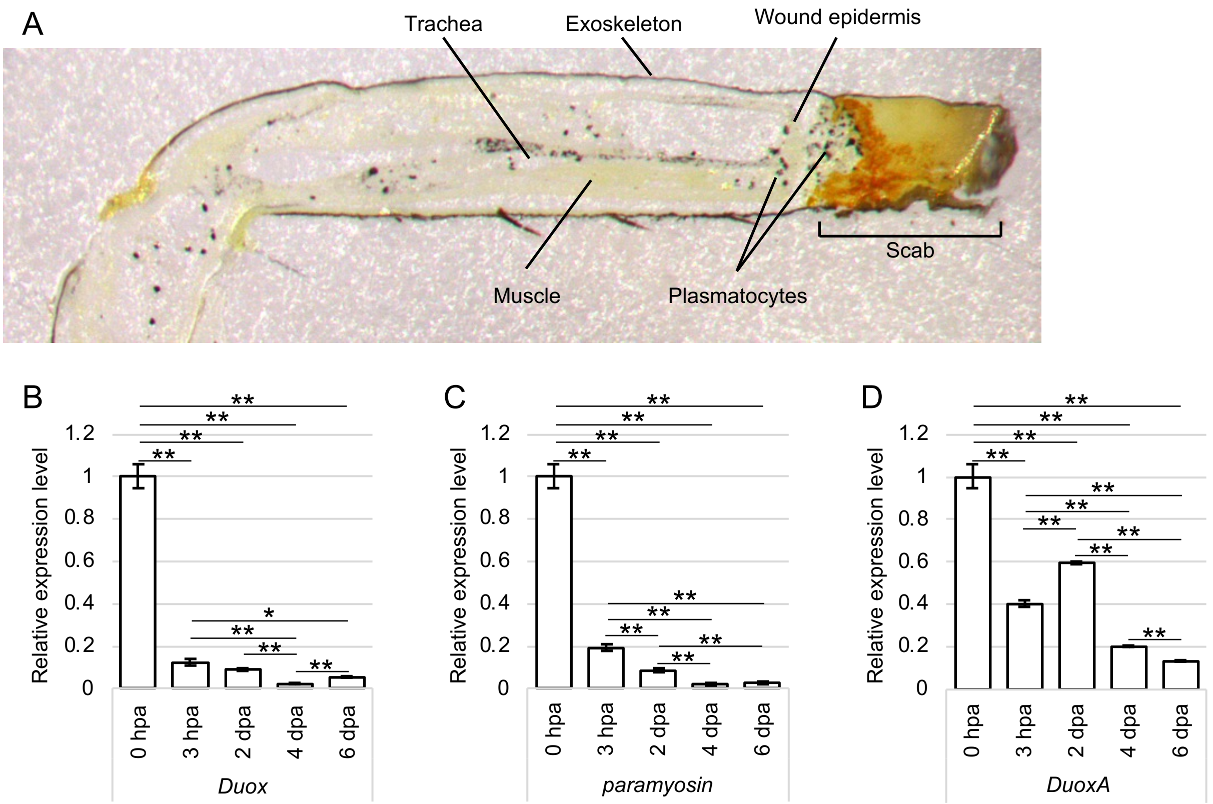

Fig. S3. Distribution of plasmatocytes and expression changes of *Duox*, *paramyosin,* and *DuoxA*. (A) Distribution of plasmatocytes was shown by India ink incorporation in the regenerating leg at 2 dpa on non-stained paraffin-embedded section. (B) Temporal expression changes of *Duox*, *paramyosin*, and *DuoxA* in regenerating legs at 0 hpa, 3 hpa, 2dpa, 4 dpa, and 6 dpa. **P<0.01, *P<0.05 (Tukey test, comparison between samples collected at each time point).

Development • Supplementary information

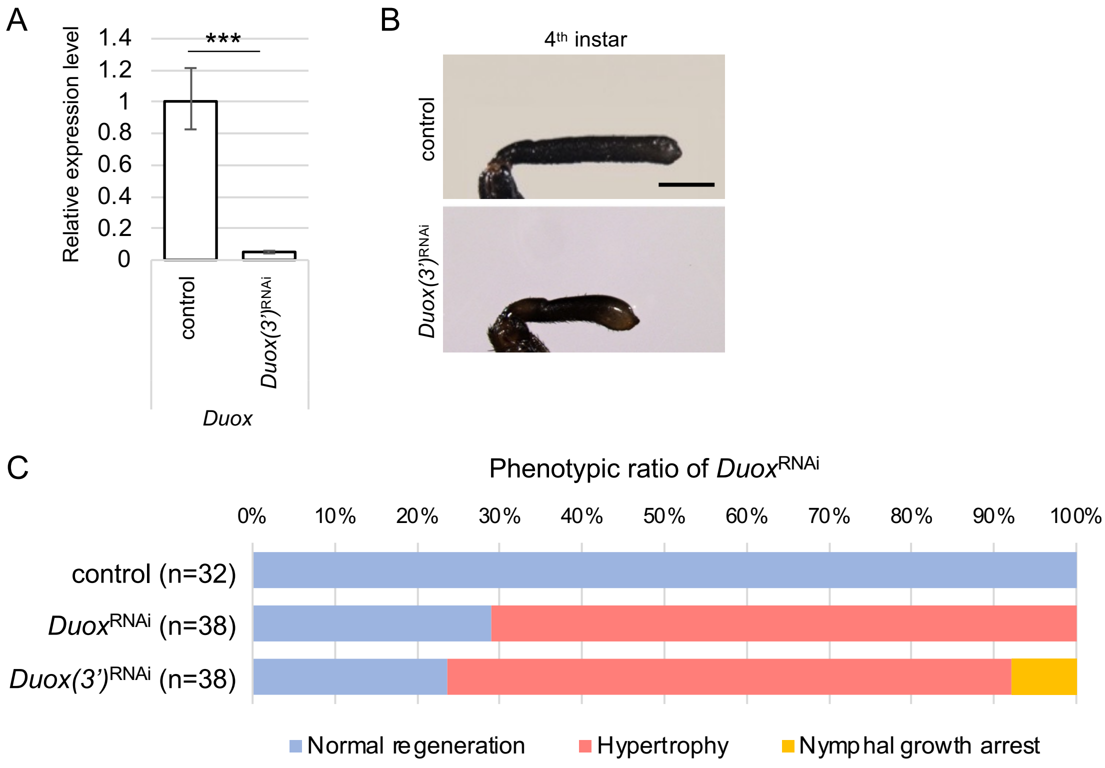

Fig. S4. Typical phenotype of *Duox(3')*^{RNAi} regenerating legs.

(A) Relative *Duox* transcript levels in the control and *Duox(3')*^{RNAi} nymphs at 2 dpa. The dsRNA region for *Duox(3')* was shown in Fig. 1E. ***P<0.001 (unpaired, two-tailed Student's t-test between control and *Duox(3)*^{RNAi}) (B) Typical phenotypes of the control and *Duox(3')*^{RNAi} regenerating legs at the fourth instar. Scale bar: 500 µm. (C) Phenotypic ratios of the control, *Duox*^{RNAi} and *Duox(3')*^{RNAi} nymphs.

Development • Supplementary information

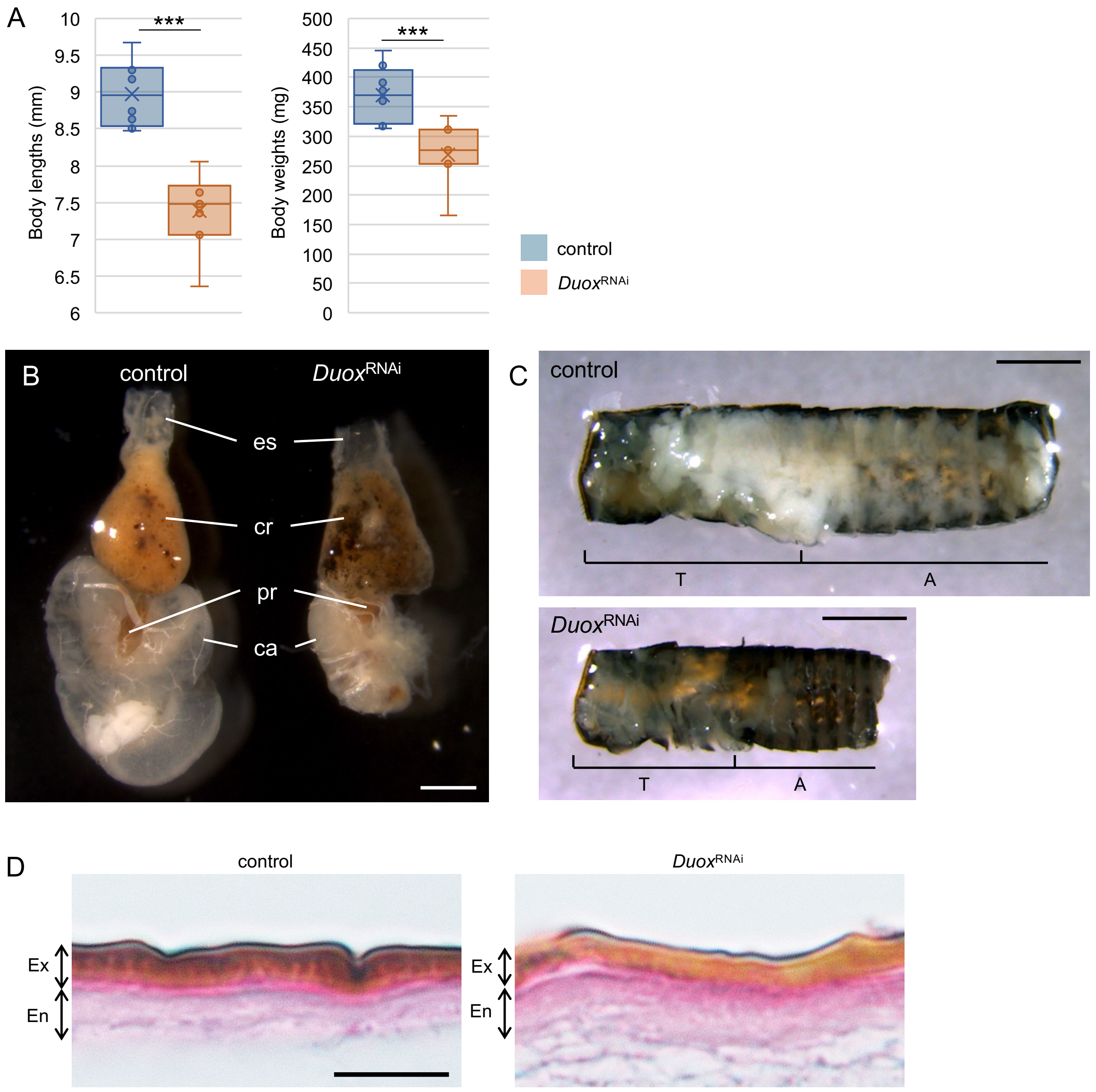

Fig. S5. Typical phenotypes of digestive organs, fat bodies, and cuticles.

(A) Body lengths and body weights of the control and *Duox*^{RNAi} nymphs at 5 dpa were shown in box charts. ***P<0.001 (unpaired, two-tailed Student's t-test between control and *Duox*^{RNAi}) (B) Dorsal views of the digestive organs from esophagus (es), crop (cr), proventriculus (pr), and caecum (ca) of the control and *Duox*^{RNAi} nymphs at 5 dpa, using a dissection microscopy. Scale bar: 1 mm. (C) The inner views of the dorsolateral exoskeletons of the control and *Duox*^{RNAi} nymphs at 5 dpa. Anterior is to the left and dorsal is to the top. T; thorax, A; abdomen. Scale bars: 1 mm. (D) High magnification images of Haematoxylin-Eosin (H-E)-stained sections of exoskeletons of the control and *Duox*^{RNAi} nymphs at 5 dpa are shown. Double-headed arrows indicate outer cuticle layer exocuticle (Ex) and inner layer endocuticle (En). Scale bars: 5 µm.

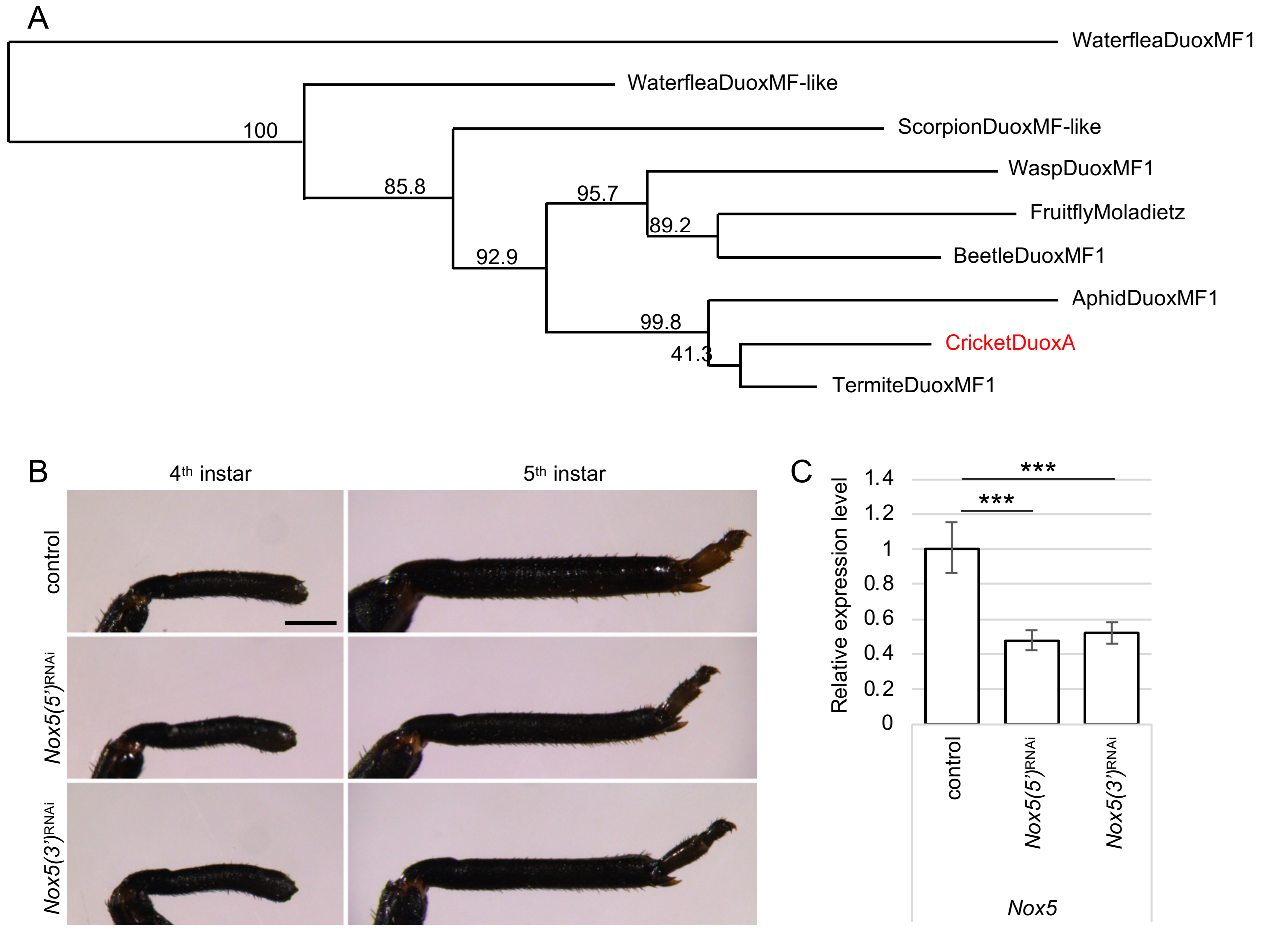

Fig. S6. Phylogenetic tree of DuoxA and typical phenotypes of *Nox5*^{RNAi}.

(A) Phylogenetic tree of insects and arthropods dual oxidase maturation factor 1 (DuoxMF/DuoxA). (B) Typical phenotypes of *Nox5(5')*^{RNAi} and *Nox5(3')*^{RNAi} regenerating legs at the fourth and fifth instar. Scale bar: 500 µm. (C) Relative *Nox5* expression levels in the control, *Nox5(5')*^{RNAi}, and *Nox5(3')*^{RNAi} nymphs at 2 dpa. The dsRNA and qPCR regions for *Nox5(5')* and *Nox5(3')* were shown in Fig. 1C. ***P<0.001 (unpaired, two-tailed Student's t-test between control and *Nox5(5')* ^{RNAi} or *Nox5(3')* ^{RNAi}).

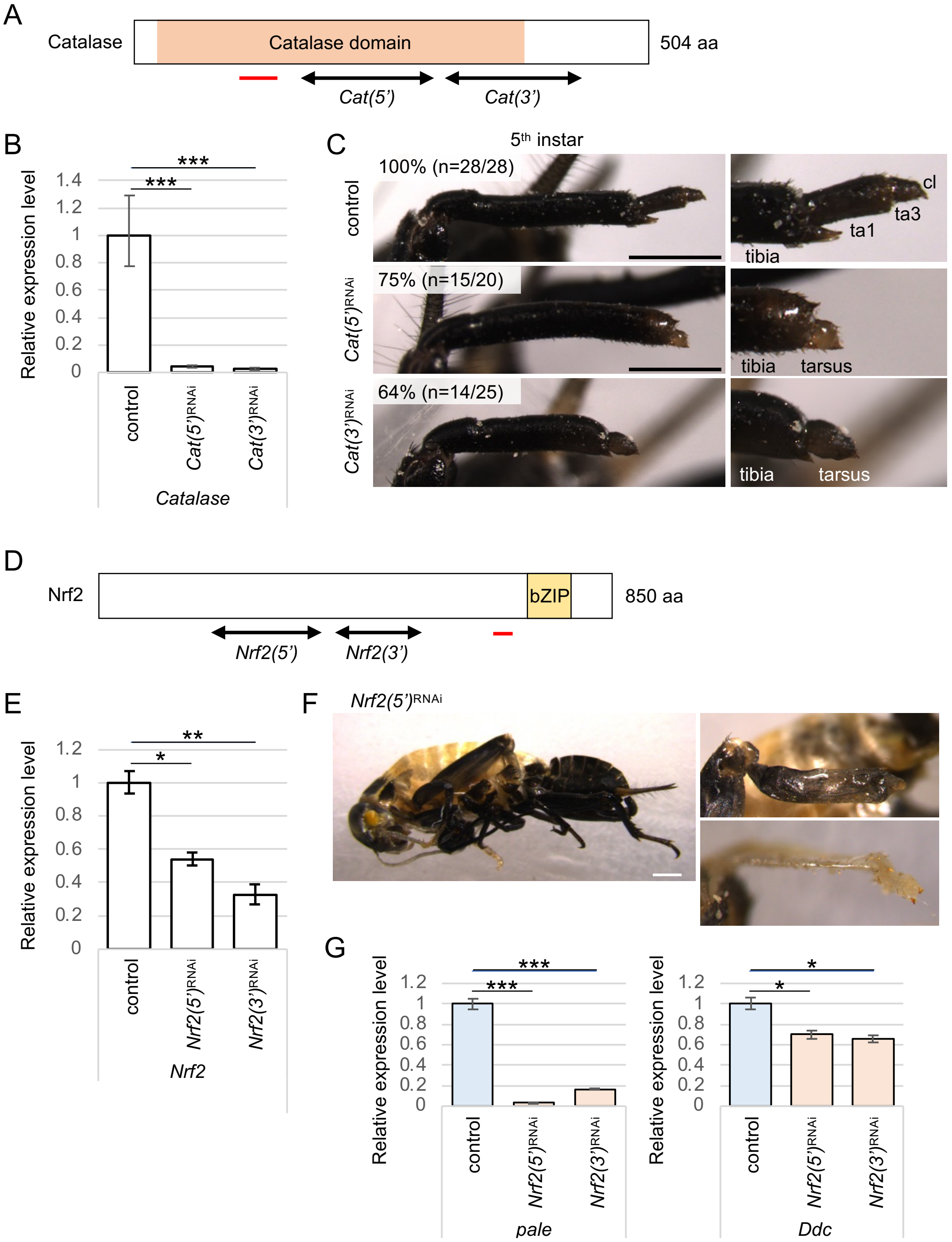

Fig. S7. Typical phenotypes of *Catalase*^{RNAi} and *Nrf2*^{RNAi}.

(A-C) Functional analysis of catalase. (A) Domain structure of *Gryllus* Catalase. Double-headed arrows and red lines indicate regions for RNAi and qPCR amplicons, respectively. (B) Relative control, *Cat(5')*^{RNAi}, and *Cat(3')*^{RNAi} expression levels determined using qPCR. ***P<0.001 (unpaired, two-tailed Student's t-test between control and *Cat(5')* ^{RNAi} or *Cat(3')* ^{RNAi}). (C) Typical phenotypes of regenerating legs at 5 dpa. Left panels are low magnification images and right panels are high magnification images of regenerated tarsi. ta, tarsal segment; cl, claw. Bars indicate 500

Development • Supplementary information

µm. (D-G) Functional analysis of Nrf2. (D) Domain structure of *Gryllus* Nrf2. Double-headed arrows and red lines indicate regions for RNAi and qPCR amplicons, respectively. (E) Relative *Nrf2* expression levels determined using qPCR. $^*P<0.05$, $^{***}P<0.001$ (unpaired, two-tailed Student's t-test between control and *Nrf2(5')* ^{RNAi} or *Nrf2(3')* ^{RNAi}). (F) Typical phenotypes of nymph and regenerating legs at 7 dpa. *Nrf2*^{RNAi} nymphs became lethal during the moult from the fourth instar to the fifth instar and inhibited melanisation. Regenerating legs of fourth instar (upper panel) and fifth instar (bottom panel) were shown. Bar indicates 500 µm. (G) Relative expression *pale* and *Ddc* levels determined using qPCR. $^*P<0.05$, $^{***}P<0.001$ (unpaired, two-tailed Student's t-test between control and *Nrf2(5')* ^{RNAi} or *Nrf2(3')* ^{RNAi}).

Development • Supplementary information

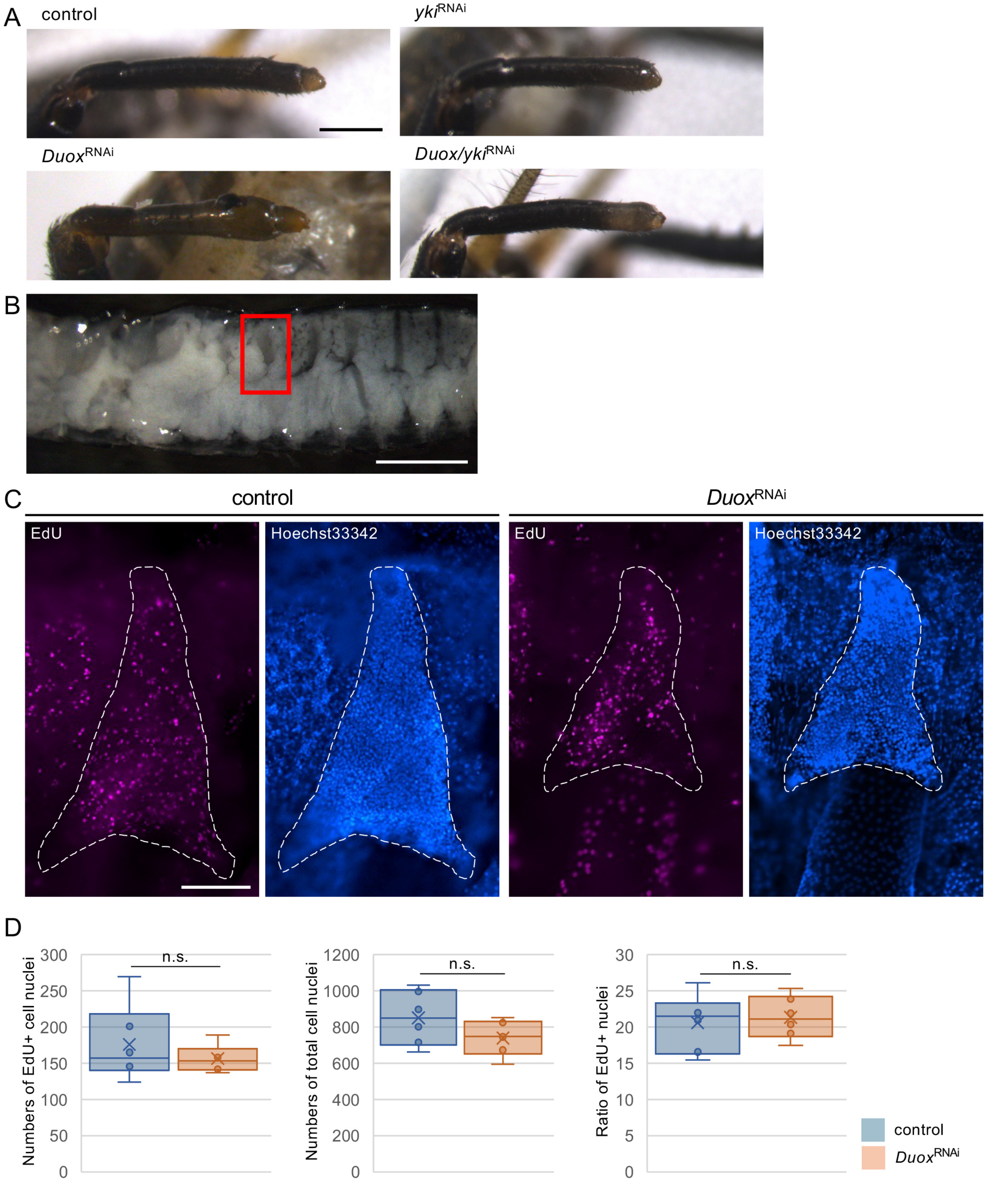

Fig. S8. Role of Yki for the hypertrophy phenotype and distribution of proliferating cells in the lymph gland.

(A) Typical phenotypes of regenerating legs of the control, *Duox*^{RNAi}, *yki*^{RNAi}, and *Duox/yki*^{RNAi} at 5 dpa. Scale bar: 500 µm. (B) The inner view of the dorsolateral exoskeleton of the control nymph at the fourth instar. The red box indicates the location of lymph gland. Scale bar: 1 mm. (C) The distribution of proliferating cell nuclei at S phase using t he EdU incorporation assay and all nuclei through

Development • Supplementary information

Hoechst 33342 staining in the lymph glands of the control and *Duox*^{RNAi} nymphs at the fourth instar. White dotted lines indicate outlines of lymph glands. Scale bar: 200 µm. (D) The number of the EdU incorporating nuclei, all nuclei, and ratios of EdU incorporating nuclei per all nuclei were shown using a box chart. No significant (n.s.) differences were noted between the control and *Duox*^{RNAi} lymph grands using the Student's t-test.

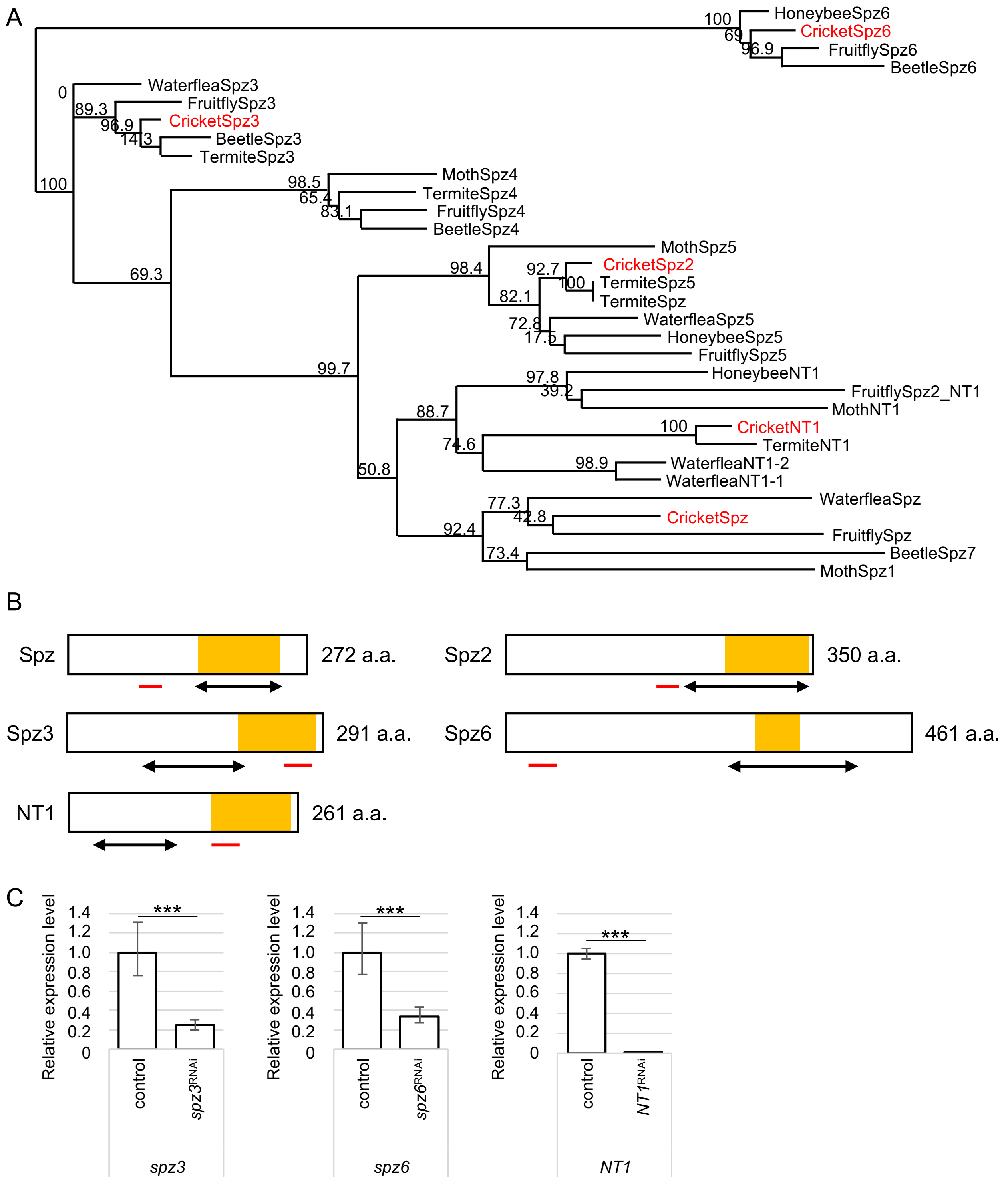

Fig. S9. Phylogenetic tree and domain structures of Spz family proteins.

(A) Phylogenetic tree of Spz family proteins of insects and arthropods on the amino acid sequence alignment using CLUSTALW. (B) Schematic diagram of *Gryllus* Spz family proteins. Yellow boxes indicate the Spaetzle super family domain. Double-headed arrows and red lines indicate regions for RNAi and qPCR amplicons, respectively. (C) RNAi efficiencies against *spz3*, *spz6*, and *NT1* are compared with the control. ***P<0.001 (unpaired, two-tailed Student's t-test between control and spz^{RNAi}, *spz2*^{RNAi} or *NT1*^{RNAi}).

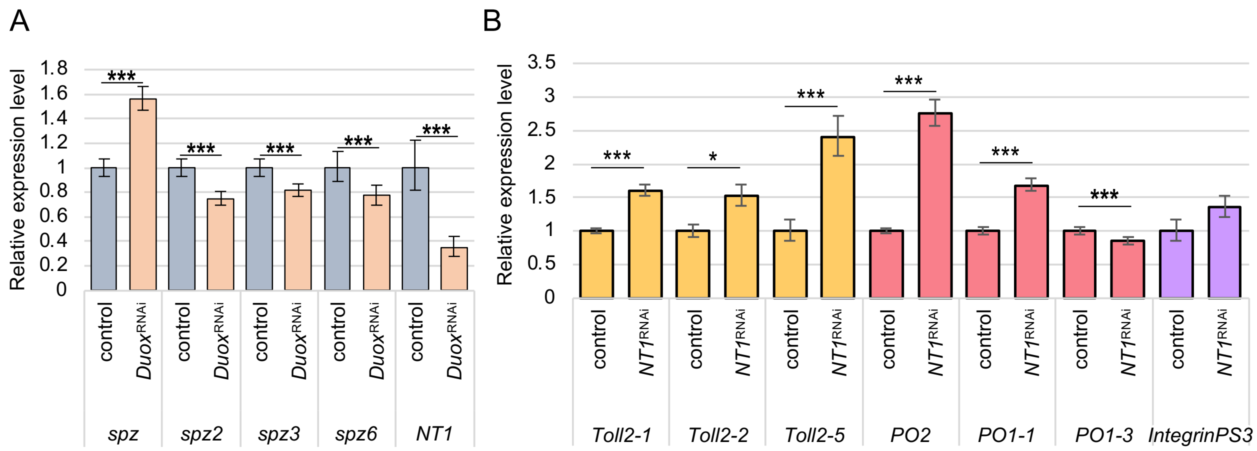

Fig. S10. Relative expression levels of Spz family and haemocytes marker genes.

(A) Relative *spz* family gene expression levels in the control and *Duox*^{RNAi} regenerating legs at 5 dpa. (B) Relative haemocyte marker gene expression levels in the control and *NT1*^{RNAi} regenerating legs at 5 dpa. *P<0.05, ***P<0.001 (unpaired, two-tailed Student's t-test between control and *Duox*^{RNAi}).

Development • Supplementary information
